# Transnational Research Networks in Chinese Scientific Production. An Investigation on Health-Industry Related Sectors

**DOI:** 10.3390/ijerph14090975

**Published:** 2017-08-29

**Authors:** Lauretta Rubini, Chiara Pollio, Marco R. Di Tommaso

**Affiliations:** Department of Economics and Management, University of Ferrara, Via Voltapaletto, 11-44121 Ferrara, Italy; chiara.pollio@unife.it (C.P.); dtmmrc@unife.it (M.R.D.T.)

**Keywords:** China, geriatrics, gerontology, research quality, network complexity, social network analysis

## Abstract

Transnational research networks (TRN) are becoming increasingly complex. Such complexity may have both positive and negative effects on the quality of research. Our work studies the evolution over time of Chinese TRN and the role of complexity on the quality of Chinese research, given the leading role this country has recently acquired in international science. We focus on the fields of geriatrics and gerontology. We build an original dataset of all scientific publications of China in these areas in 2009, 2012 and 2015, starting from the ISI Web of Knowledge (ISI WoK) database. Using Social Network Analysis (SNA), we analyze the change in scientific network structure across time. Second, we design indices to control for the different aspects of networks complexity (number of authors, country heterogeneity and institutional heterogeneity) and we perform negative binomial regressions to identify the main determinants of research quality. Our analysis shows that research networks in the field of geriatrics and gerontology have gradually become wider in terms of countries and have become more balanced. Furthermore, our results identify that different forms of complexity have different impacts on quality, including a reciprocal moderating effect. In particular, according to our analysis, research quality benefits from complex research networks both in terms of countries and of types of institutions involved, but that such networks should be “compact” in terms of number of authors. Eventually, we suggest that complexity should be carefully taken into account when designing policies aimed at enhancing the quality of research.

## 1. Introduction

In the modern age, science is characterized by a generalized enlargement of research networks. The number of authors taking part in a single publication has increased. In particular for medical sciences, we observe that the average number of authors per article in PubMed has passed from 2.0 in 1970 to 5.3 in 2010 [1]. The complexity in handling scientific cooperation networks has also been rising over the years, because cooperation increasingly takes place at transnational level [2,3,4,5,6,7]. In 2010 almost 25% of research articles included authors from at least two countries (against 10% in 1990) [8]. Such process has been called the “globalisation of science” [9]. Not only scientific cooperation networks have increasingly included several researchers from different countries. They have recently also seen the contribution of authors coming from different types of institutions, with different general goals and organization [10].

Large, multi-national and multi-institutional collaboration networks are challenging since they embed high risks and wide opportunities.

As far as risks are concerned, the early theories of collective actions suggest that, typically, large groups have more problems in cooperating because of possible free-riding, costs of coordination, monitoring and sanctioning [11]. These costs might increase for scientific cooperation involving distant disciplinary fields, even if it has also been said that this kind of interaction might be a fertile soil for the emergence of radically new ideas [12]. Other costs (in terms of funds and time) might be due to the mobility of equipment and personnel, but also to the necessity of preparing, defining and managing the agreement or of coordinating all the actors involved. There might be difficulties in putting together different cultures, financial and reward systems, and so on [13]. The smooth functioning of networks furthermore requires that the intention distance among actors in terms of goal is not too large [14]. Indeed, intention distance and network complexity can mutually reinforce each other, as with the growth in the number and variety of actors participating in the network, it is more likely that goals diverge [15]. In this framework, a university-to-university relation might be somewhat easier to manage than a relation involving firms, since partners operate in similar institutions and share the same set of values. With different institutional partners there might instead be different positions regarding the disclosure of the results, different priorities due to different career paths or different degrees of intensity in the effort put in the relationship [16].

Transnational research networks (TRNs from now on) may incur in even higher difficulties when they involve other institutions. This is the case of TRN aimed at the production of health knowledge. In fact, this field involves public as well as private producers, collective entities as well as individuals, profit oriented as well as non-profit organisations (i.e., hospitals, laboratories, physicians, etc.). It is very likely, therefore, that each category has a different main goal (profit maximization, provision of the service per se, societal wellbeing etc.) that leads to a different interpretation of the role that every actor is supposed to have in the sector. Moreover, further sources of complexity are related to the nature of the knowledge produced in the health sector, which usually requires high-tech devices and techniques and the ability to handle them, as well as the capacity to meet high quality standards that require rigorous quality controls. Consequently, participants in knowledge co-production networks in this sector are required to have high and flexible technical knowhow and knowledge skills.

The complexity of networking in these contexts may translate into a higher degree of fragility of the relation. Networks might be destined to fail due to relevant information asymmetries, wide free-riding opportunities, and potential conflicts in defining rules, goals and strategies.

However, in present times complex transnational networking in co-production of knowledge—also and in particular in the health sector—is seen by many as highly desirable. Cooperation allows to have access to new expertise and complex equipment, due to the growing complexity of science; increases the efficiency and speed of the research work, thanks to the possibility of counting on a larger team; facilitates the study of more complex and multidisciplinary problems; allows a cross fertilization of competences that facilitates the birth of new ideas; gives scientists the possibility to directly learn new competences by interacting with others, reducing isolation; makes the research work more pleasant and stimulating, by giving to people the possibility to interact with others and to travel; and improves the accessibility to funds. The consequence is an increase in prestige and visibility of participants, who can multiply the possibility to get in touch with other colleagues, and to enhance their professional status. Studies show in fact that collaboration can result in a higher rate of acceptance for publication from prestigious journals, in a higher academic productivity (in terms, for example, of number of published papers) and in a higher quality of research [4,6,13,16,17,18,19,20,21]. On the contrary, researchers working in isolation are less productive, less informed and consequently less acknowledged [22]. Co-research ultimately contributes to the scientific progress, allowing to combine and integrate competences, to ignite cross-fertilization procedures and to favor the interchange of experiences [23,24].

In the broad scenario of transnational research cooperation, China is one of the most interesting cases. Starting from the launch of the open door policy, China has put it at the center of its scientific and technological development, as a key factor to favor the modernization of the country [25,26]. Consequently, its position within the international scientific scenario has been constantly increasing over the years [27]. Furthermore, the national scientific community is increasingly open to international partnerships. Up to the eighties, the number of internationally co-authored papers of scientists located in China was very limited, since in the pre-reform period only a few selected scholars were allowed to publish in international journals, and they were strictly controlled. Afterwards, the government has constantly increased its commitment towards the improvement of international collaboration in science and technology, with the first boost given by the S&T Cooperation Program for Priority Projects launched with the 10th Five-Year Plan 2000–2005. Since then, all the Chinese research institutions, including universities, have increased the programs and incentives to international collaboration and there has been a constant increase in the number of internationally co-authored papers in the country [28]. Such increase may be explained by the opportunity, given to Chinese researchers by international collaborations, to access advanced knowledge and instruments, and enter more visible circuits, therefore enhancing their potential impact [29].

The habit of carrying out collaborative research within transnational research networks is much more common in health-related fields than in other scientific disciplines. Currently, around 50% of articles published by Chinese scientists in ISI-Web of Science journals on health issues were characterized by collaborations with foreign scientists, against a much lower 20% of the total Chinese publications.

In this framework, we believe it is particularly interesting to deepen our knowledge on the evolution over time of TRN involving Chinese scholars and on the factors affecting the quality of research carried out in health-related fields, with particular reference to the impact that complexity of TRN may have. To do so, the study focuses on the field of geriatrics and gerontology (hereinafter gerontology). This specific field has been chosen for several reasons:

(1) Given the current effects that 30 years of one-child policy are showing on the population aging, the field is increasingly popular among scientists (and policy makers), as ageing of Chinese population is becoming a growing concern [30,31,32];
(2) Studying a very specific field allows to avoid comparisons among different sectors, that might have different behaviors in terms of co-publishing and citations [33,34,35];
(3) The number of papers produced in the selected field, despite the growing popularity, is still small enough to allow to collect the detailed information that is needed to perform the study across time. At the same time, numbers are high enough to apply an econometric model sufficiently reliable.

## 2. Materials and Methods

### 2.1. Data Collection

The main aim of our empirical study is to show the changes occurred over time in Chinese TRN in gerontology and to identify the determinants of research quality, with particular reference to the effects of complexity.

In this paper, we have chosen to measure the quality of science by means of the number of citations received by a publication. We are aware that the use of this indicator as a proxy for the quality of a published paper is quite controversial in the literature. The use of citations is based on the assumption that authors select the works to cite on the basis of their quality: therefore the most cited articles are those with a higher quality level [36]. However, since the motivations to cite a paper are actually much more varied, several distortions might arise. Among these:

- A paper might have a high citation rate not only when it is highly appreciated, but also if it is greatly criticized [34];
- a paper by an influential scientist may be more visible even if its quality is no greater than other works [34];
- self-citations might artificially distort the number of citations [37,38];
- low citation rates might simply be the consequence of a narrow or unpopular research field rather than of poor quality [39,40];
- the number of citations not only depends on the quality of the article, but also on the location of the author and on the prestige, language and diffusion of the journal [34,40].

In spite of these limitations, the use of citation count is mostly supported ([39,40,41,42], only to cite some). In particular, it has been asserted that, with sufficiently large numbers, the different reasons for citing neutralize each other [43] and that there is “sufficient evidence that (the different) ‘reference motives’ are not so different or ‘randomly’ given to such an extent that the phenomenon of citation would lose its role as a reliable measure of impact” [44] (p. 26). The validity of citations as proxy of the quality of published works is confirmed by the fact that is has shown a strong correlation with several other indicators, such as Nobel Prize, honorific awards, number of publications, grants awarded to a department and peer-review assessment [18,45,46,47]. Considering that—as suggested in the literature:

1. we analyze a very specific sub-field [34,35];
2. in this scientific field, publication in international journals is one of the most utilized means of diffusion of results [4,48] and
3. we only take into account English publications in order to avoid language bias, we believe that, for our analysis, citation count can be considered a reliable proxy of the quality of the underlying research in a publication.

To perform our study, we have built a unique dataset including 1381 scientific publications of China-based scholars in the field of gerontology and geriatrics available on ISI Web of Knowledge (from now on referred as ISI WoK), developed by Thomson Reuters and internationally acknowledged as the one including at present the most prestigious scientific journals, covering over 8000 scientific journals worldwide in sciences, social sciences, arts, and humanities [49]. We have collected data for the years 2000 (18 publications), 2009 (154), 2012 (346) and 2015 (865). Due to the low number of publications in 2000 we have used the information only for the Social Network Analysis (SNA), while we have excluded them from the descriptive and the econometric analyses.

Our database contains a broad range of information on each publication. Some were readily available from ISI WoK, and concern our outcome of interest (the number of citations up to May 2017), together with some control variables (the journal of publication and the main sector(s)). However, a large part of our dataset has been built upon a careful content analysis of each publication, allowing us to extract the number of authors participating in the publications, the typology of institution of affiliation (which we classified into university, hospital, university-hospital, company, association, and public institution), the country of each institution, and the country and institutional affiliation of the reprint author. Such hand-collected information is the core of our analysis and constitutes the basis for building our main variables of interest.

### 2.2. Data Analysis

#### 2.2.1. Social Network Analysis

A first set of information can be obtained by dividing our sample between publications authored only by scholars based in China (“China-only” publications from now on), and those co-authored by international and Chinese scientists (“international” from now on).

**Figure 1:**
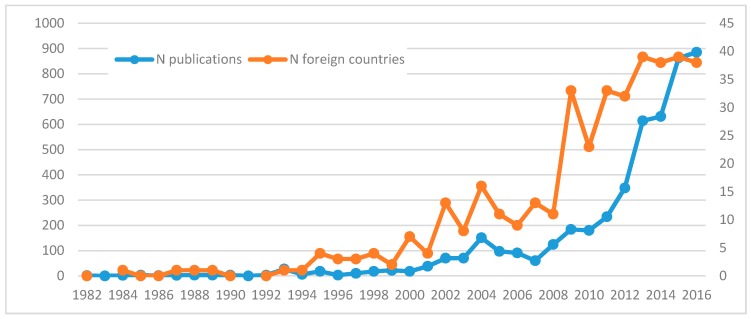
Evolution in the total number of Chinese publications and total number of countries involved in the field of gerontology. Source: authors’ elaboration based on ISI Web of Knowledge ISI WoK data.

Analogously to the whole Chinese scientific production, the number of publications by Chinese authors in the ISI WoK database has risen exponentially over the years also in the case of gerontology and geriatrics. This surge has started in 2000, but it has acquired an unprecedented speed from 2008 on. At the same time, China has increased the size of its TRN, which was virtually non-existing until 2000. In parallel with the number of publications, the number of countries has increased particularly from 2008, and in more recent years it has arrived to include about 40 different countries (Figure 1).

**Figure 2:**
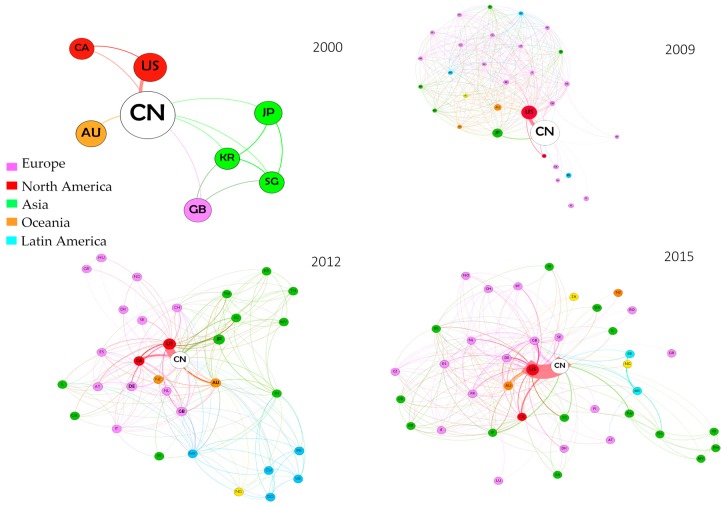
Chinese transnational research networks (TRN)s in gerontology. Source: authors’ elaboration based on ISI WoK data. Note: the dimension of nodes is proportional to the times in which the country hosts the reprint author while the width of the linkage is proportional to the number of publications involving the two countries.

However, the TRN did not change only in terms of number of countries involved, but also in terms of relations within the groups of partners, weight of the relations and direction of the network. We have studied all such evolutions by means of a Social Network Analysis (SNA), whose results are presented in Figure 2. In each of the graphs representing each year, the dimension of nodes is proportional to the times in which the country hosts the reprint author while the width of the linkage is proportional to the number of publications involving the two countries.

In 2000 the gerontology-geriatrics TRN was very basic, with only seven foreign countries involved and with China coordinating most of the international publications (the relative size of China’s node is in fact much larger than the others). Already in this initial phase of internationalization, China shows a preferential relation with the USA, deductible from the thicker link between the two countries. Very marginal in this first stage is the role played by Europe, with only one connection to Great Britain.

Over the years the number of countries involved has increased, the relation with the USA has become tighter and tighter, while at the same time the relative role of China as guide of the network has decreased. The peculiar shape of the 2009 network is due to one publication involving almost all countries, and this justifies the fact that the TRN in that year is particularly dense, because all countries in the group are connected with one another by means of the common publication. Passing instead from 2012 to 2015, the network has become less dense and compact. While in 2012 relations with other countries were mostly intra-continental, in 2015 the network has reached a higher degree of “inter-continentality”. There seems to be a higher number of relations involving countries belonging to different geographic groupings, while China maintains relatively more isolated relations only with a small number of countries (such as Greece or Luxembourg). There still seems to be a sort of close “continental” relation only with some Asian countries—namely Thailand, Indonesia, Malaysia and the Philippines—that are connected almost exclusively to one another. The dominant bilateral relation remains that with the United States, which seems to become stronger and stronger over time. As regards the guidance of TRNs, China still holds the reins in the majority of cases.

#### 2.2.2. Descriptive Analysis

**Figure 3:**
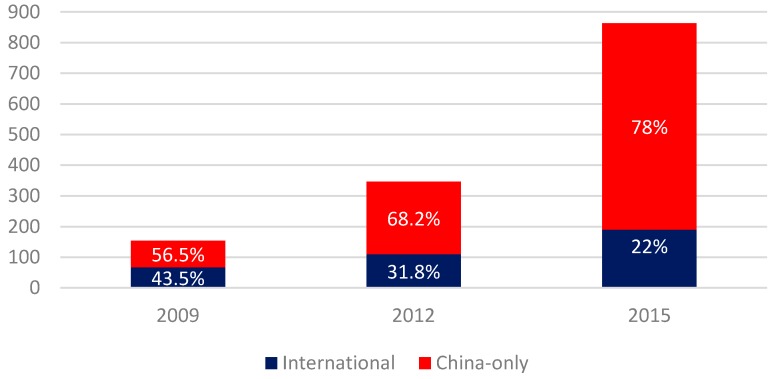
International vs. China-only publications. Number and percentage. Source: authors’ elaboration based on ISI WoK data.

As a whole, there are 996 out of 1363 publications that are co-authored only by China-based authors and 367 that are co-authored by foreign authors as well. As Figure 3 shows, the absolute number of international publications has certainly grown across time. However, the gap between international and China-only publications has widened as well: if in 2009 the number of China-only publications accounted for slightly more than one half than the total of publications, in 2015 the situation had radically changed, as the international publications accounted only for the 22 percent (Figure 3).

Within the international networks, the role that China plays may vary greatly. In particular, we distinguish among six possible cases, according to the weight of Chinese institutions on the total and the nationality of the reprint author. With regard to the role of China in terms of number, we identify three cases: (1) when Chinese institutions are more than 50% of the total (majority); (2) when Chinese institutions are less than 50% of the total (minority) and (3) when Chinese institutions are 50% of the total (parity). In terms of the reprint author, it can be either Chinese or not (either foreign or not available in the publication). The distribution of publications across the six categories is represented in Table 1.

The pool of 367 international publications distributes evenly across those where Chinese actors are the majority (128), those where they are the minority (134) and those where they are exactly the 50% of participants (105). At the same time, Chinese authors coordinate approximately the same number of networks than their foreign colleagues. However, once we combine number and reprint variables, further interesting evidence emerges. About 16 percent of the publications do not have a leading author, and this occurs mainly for the publications where there is a parity of Chinese and foreign authors. Chinese authors lead about the same number of publications than their foreign colleagues, and they distribute in a mirroring way: in other words, Chinese are reprint authors mainly when they are the majority of the collaborating members, while foreign authors lead the network when Chinese partners are a minority. Overall, the matrix shows a rather balanced distribution when we look at variables one by one. However, we find a rather polarized situation—between a Chinese-majority-Chinese-led group of publications and a Chinese-minority-foreign-led group of publications—when we analyze them jointly.

#### 2.2.3. Complexity Indices

To study the way in which complexity affects the quality of research, we identified three dimensions of network complexity: number of authors, institutional heterogeneity and country heterogeneity. The first is measured directly through the number of co-authors in a single publication. In order to measure institutional and country heterogeneity, we summarized the information for each dimension through a normalized Shannon’s index of entropy [50,51].The Shannon or entropy index is defined as:

(1) $$S_{i}=-\overset{n}{\underset{i=1}{\sum }}p_{i}\ln p_{i}$$

where *p_i_* is the probability of each event in the set H = *p*_1_, *p*_2_ … *p_n_* to occur. It takes the largest value when all the events of the set have the same probability to occur, and the lowest when only one event has the probability to occur. We exploit this feature to study how heterogeneous our phenomenon of interest is: that is, if the index is large, we consider that the publication has a higher degree of countries’/institutions heterogeneity, being their distribution less concentrated in one or few categories and more spread across various types of countries/institutions. We standardized both the Shannon index for countries heterogeneity *S*(*c*) and the Shannon index for institutional heterogeneity *S*(*i*) to allow them to vary between 0 (minimum heterogeneity) and 100 (maximum heterogeneity). For country heterogeneity, *S*(*c*) takes value 0 when no foreign countries collaborate with China; it takes value 100 when the number of foreign collaborating countries is the highest in our sample. With respect to the institutional heterogeneity index *S*(*i*), we have minimum heterogeneity when only a type of institution is taking part in the publication (irrespective of the number of institutions). *S*(*i*) takes value 100 when all the 6 types of institutions are participating in the same proportion to the publication. Finally, we also standardized the number of authors to obtain a variable (*STD_AUTH*) whose range is between 0 (minimum number of authors) and 100 (maximum number of authors in the sample).

**Figure 4:**
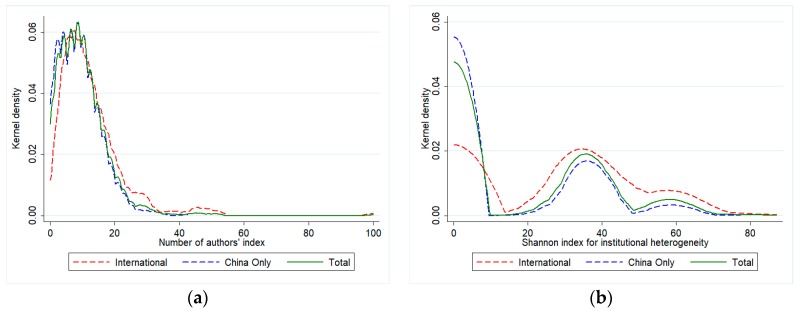
Distribution of index complexity indices for International and China-only publications: (**a**) Number of authors’ index, kernel estimates; (**b**) Shannon index for institutional heterogeneity, kernel estimates. Source: authors’ elaboration.

**Figure 5:**
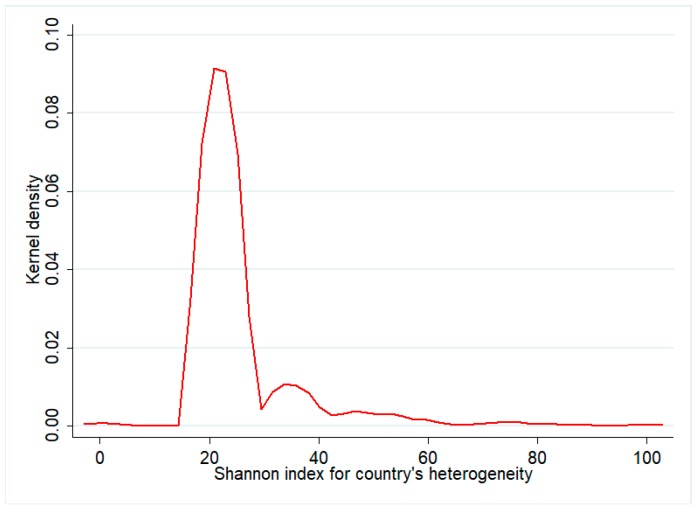
Shannon index for country’s heterogeneity for International publications, kernel estimate. Source: authors’ elaboration.

In Figure 4 and Figure 5 we report the kernel distributions of the variables representing the dimensions of complexity that we take into account. For the index of number of authors and the Shannon index for institutional heterogeneity (Figure 2), we decompose the distribution of the total of publications (in green) in that of the international (red) and China-only (blue) groups. Although the position of the distribution for international publications is slightly more to the right, the distribution of the number of authors’ index for the two groups seems to be similar, as the largest part of the observations is concentrated in all cases around a low value of the index (equal to 10). There are more differences in the kernel distributions of the Shannon index of institutional heterogeneity: in this case, it is clear that while China-only publications are largely homogeneous in terms of types of institutions taking part in the publication, a larger variety is observable in the international collaborations. Finally, Figure 5 only shows the kernel density for International publications, as for the China-only group the value of the Shannon index is, by construction, always 0. For such index, international publications are mainly distributed across an interval of medium-to-low values, between 20 and 40. From this visual analysis, we may claim that international and China-only publications tend to be rather similar in terms of number of authors, but different in terms of institutional heterogeneity.

#### 2.2.4. Parametric Estimation

To see the effects of complexity on the quality of publications, we parametrically estimated the impacts in a model when the number of citations is a function of a set of variables. For each *i* publication, we have:

*CIT*_i_ = f(**S**_i_, **C**_i_, **X**_i_) (2)

where **S**_i_ is the vector of the complexity indices related to the number of authors (*STD_AUTH*), number of countries (*S*(*c*)) and number and types of institutions (*S*(*i*) participating in the publication. The vector **C**_i_ includes two variables that measure the impact of Chinese coordination of the network. With *CN_repr_maj*_i_, we wish to capture whether there exists an effect of Chinese actors being both the majority of participants and the coordinators of the network. With *CN_repr_min*_i_, we want to assess the effect of Chinese actors being the coordinators of the network when they are a minority. The third case when a Chinese is a reprint author—parity between Chinese and foreign actors—was excluded due to collinearity. A description of these two variables is presented in Table 2. Finally, the vector **X**_i_ includes other control variables that are summarized in Table 2, while a correlation table for all independent variables is reported in the Appendix (Table A1). With the variable *Nr_Sect* we wish to capture the degree of interdisciplinarity of the publication. *Repr_NA*, *Repr_UNI*, *Repr_FOR* and *Repr_US* are all dummies related to the reprint authors: not available, affiliated to a university, foreign, from the US *ASS*, *COMP*, *HOSP*, *PUB*, *UNIHOSP* and *UNI* are dummies representing whether at least one association, company, hospital, public institution, university-hospital and/or university takes part in the publication. They are not exclusive to one another, as in the same publication there could be both at least one hospital (*HOSP* = 1) and at least one company (*COMP* = 1) and so on. Additionally, we include a dummy that captures the participation of a US-affiliated academic, given that this country has a prominent role in the publication network of China, as the SNA showed. Finally, in order to control for the quality of the journal of the publication we include *Impact_Factor*, which is the 5-year impact factor of the academic journal. Year dummies are also included in all the specifications.

Due to the features of the variable representing the number of citations, linear models would not fit in this case. In fact, our outcome of interest *CIT* is a count variable with a largely asymmetric distribution, and for a large part of observations the value is zero (Table 3). The skewed distribution and overdispersion of the dependent variable *CIT* suggest the use of a negative binomial model [52,53,54].

In the next section we perform three regressions. The first represents the baseline model, in which we analyze the effect of all the variables on *CIT* taking into account all publications. In the second specification, we wish to identify whether any complementarities or substitution effects exist among the three dimensions of complexity (number, countries heterogeneity, institutional heterogeneity); therefore we add to the baseline model three two-way interactive terms among the three variables. In the third regressions we finally add the two variables that allow us to analyze the impact of the management of the network and of the role that China plays within the network.

## 3. Results and Discussion

Table 4 shows the results for the negative binomial regression of the three specifications of the model we identified previously, while Table 5 reports the average marginal effects. Columns 1 in both tables report the results for the baseline model. Concerning our three dimensions of complexity, the *institutional heterogeneity* does not affect quality, while *country’s heterogeneity* is significant (and negative) only in the second formulation, when we introduce the interaction terms. However, such negative effect has a rather limited magnitude (−0.09 citations every increase of 1 point in the index). The only dimension of complexity that seems to affect—positively—the quality of publications in all three formulations is the standardized *number of authors*. However, this is not sufficient to assert that larger co-authorship networks translate in better research. With a higher number of authors, in fact, the number of citations might increase simply because there are more possibilities for the article to be found and read. This is because each of the authors not only might have his/her own citation rings, but is also simply another possible keyword to be used to search for the article. More information on the role of this variable will be deduced by the use of interaction terms in the second formulation of the model.

As far as the other determinants of research quality are concerned, the three formulations provide the same results for some variables:

- *The degree of interdisciplinarity of the publication seems to negatively affect its quality*. In fact, the number of sectors to which the publication refers has a negative impact on the number of citations;
- *The absence of a reprint author noticeably decreases the quality of publications*, causing a reduction in the number of citations by 29.7 in comparison with articles with the indication of the reprint author. We have considered the corresponding author as the person in charge of maintaining the relation with the journal and with the remaining actors in the partnership, therefore acting as the one de facto managing the relation. Even if it is not clear why some publications do not mention any reprint author, the effect that the lack of a direction has on the research quality seems to be evidently negative;
- *The participation of US institutions in the partnership always has a positive impact on citations*, which increase—due to this participation—by a range that goes from 2 to 2.5, depending on the specification, as shown in Table 5. Other formulations of our model have been tested including other countries, such as those belonging to the G7 group, but no one has resulted to have an impact, apart from the USA. It is possible to read this result both as an effect of the possibility of accessing high tech research laboratories, world-renowned scientists and top scientific circuits, but also simply as a consequence of a higher visibility of US scientists and of a common habit of Americans to cite each other. Undoubtedly, at present the scientific world is US-centered, and our results confirm that being able to be part in this world allows to increase the visibility (and possibly the quality) of the carried out research.
- *To publish in journals with a high impact factor raises the number of received citations.* This might occur because publishing a paper in a prestigious journal (i.e., highly ranked in terms of Impact Factor) per se increases the possibilities to receive a high number of citations. However, this should not be read as a weakness in the use of citation counts for the measurement of quality. On the contrary, considering that journals included in the impact factor system are all peer-reviewed, it seems to confirm the fact that a positive assessment from peers anticipates an equally positive assessment from the scientific audience.
- *The presence of universities in the partnership also increases citations.* This aspect might be simply related to the fact that to cite previous works is particularly diffused within academic circuits. For analogous reasons also university-hospitals have a positive impact, but only in the first formulation.

As far as *interactions* are concerned, the effect of the one between countries and institutional complexity indices is positive, while that between institutional heterogeneity and number of authors is negative. However, a correct interpretation of the meaning of the interactive terms requires some specifications. Because marginal effects of each variable are computed keeping all other variables fixed, a correct estimate of such effects should not include the values for interaction terms [55]. This is because, by definition, the value of the interaction is dependent on the value assumed by its components, so its marginal effect on the outcome of interest cannot change independently on them.

**Figure 6:**
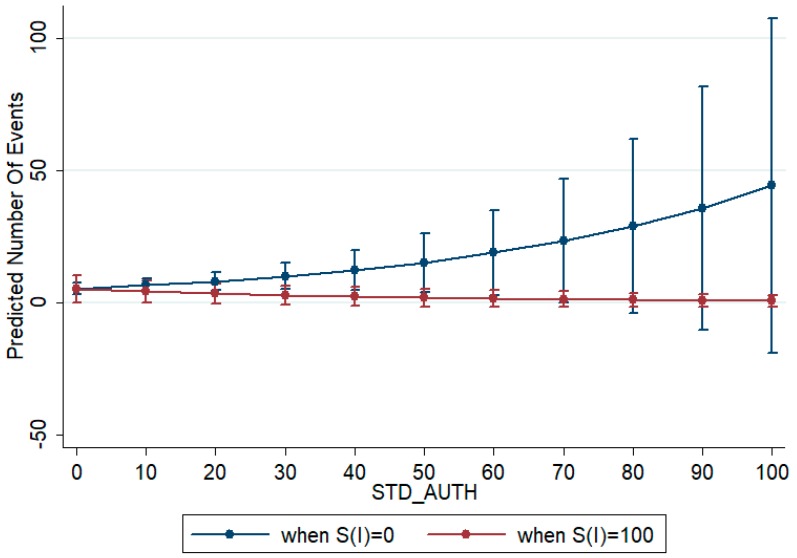
Effect of number of authors’ index on citations when institutional complexity is minimum (*S(i)* = 0) and when institutional complexity is maximum (*S(i)* = 100). Source: authors’ elaboration.

**Figure 7:**
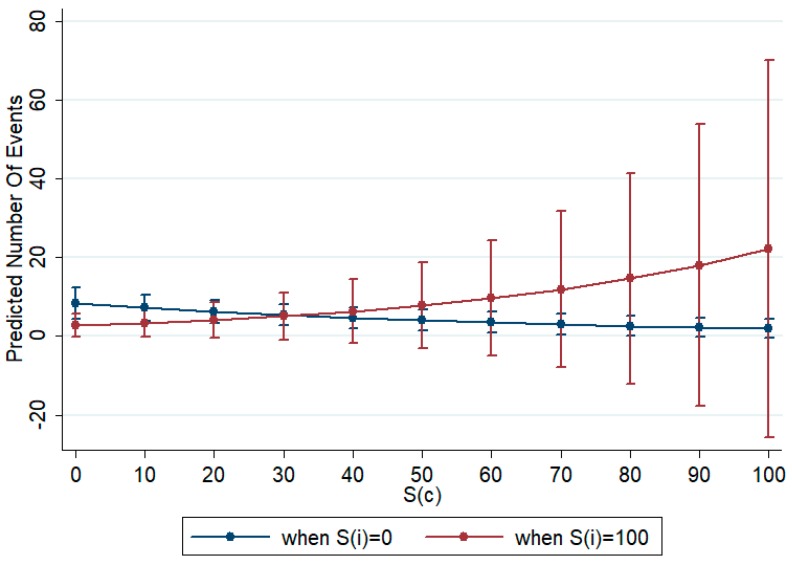
Effect of number of country’s complexity index on citations when institutional complexity is minimum (*S(i)* = 0) and when institutional complexity is maximum (*S(i)* = 100). Source: authors’ elaboration.

To understand whether the interactions between the complexity indices amplify or reduce quality, we graphically show their covariation by fixing them at specific values. In this case, we chose to analyze how indices covariate when complexity is at its minimum and its maximum. In Figure 6 and Figure 7 we show the variation in the number of citations (predicted number of events) related to the standardized number of authors (STD_AUTH) and to countries’ complexity (*S(c)*) when the institutional complexity, measured by *S(i)*, takes the minimum (0) or the maximum (100) value. From the analysis of the two graphs, we can identify a large moderating effect of institutional complexity on the positive effect of number of citations and of the negative impact of country’s complexity on quality. In the first case (Figure 6), when institutional complexity is equal to 0, if the number of authors increases there is a positive impact on quality. However, if institutional complexity is at its maximum, to enlarge the network by increasing the number of authors has a depressive effect on quality. In the second case (Figure 7), if the research network is homogeneous in terms of institutions (*S(i)* = 0), to increase the number of countries induces a decrease in quality. When instead the institutional complexity is maximum, to have larger networks in terms of countries involved has a positive impact on quality.

Another interesting aspect of our results emerges in relation with the *role that China plays* within the network. When we include the variables representing the role of China as a majority or minority and as reprint author, we observe a negative effect of Chinese institutions as reprint author when they are a minority (about 5.5 citations less), while no effect is significant when they are a majority. One possible interpretation of the first result is that, when Chinese institutions are the minority within the network, the coordination of the partnership is entrusted to a Chinese author only for low quality research. Vice versa, in cases in which Chinese institutions are the majority, there is no impact on quality of the nationality of the reprint author. This means that China’s coordination of such networks has the same impact on quality than if the coordination is assigned to foreign institutions.

## 4. Conclusions

The analysis carried out in this work had the aim to shed some light on the evolution over time of Chinese TRN in gerontology and on the possible determinants of research quality in the field. By means of SNA we have seen that over a time span of only 15 years, the TRN that China has been able to build in this scientific sector has become wider, more complex and diversified, but also still under the main control of Chinese institutions. This has induced us to see if these changes have had an impact on the quality of the research itself.

To explore this aspect we have designed a negative binomial regression whose results have allowed us to identify some determinants in the quality of research and to clarify the role that complexity has on research quality in gerontology. Our findings suggest that, ideally, in order to have the maximum impact on the number of received citations, and therefore on quality, research in gerontology should be carried out on very specialized issues, involving a small number of scientific sectors. The network should include one or more US institutions, and at least one university. Furthermore, we have observed that those articles published on high impact factor journals tend to be cited more. Regarding the actors coordinating the network, a leading role assigned to China has a negative impact on quality only in cases in which the majority of the institutions involved in the partnership are foreign.

The investigation of the impact of complexity on quality has been carried out by means of three different measures of complexity: (1) size complexity; (2) institutional complexity; (3) country complexity. We have studied their separated effect on research quality and we have used interaction terms in order to capture their joint effects. Our results indicate that complexity does play a role in affecting quality of research carried out in collaboration.

More interestingly, our study suggests that there exists an interaction effect among the various dimensions of network complexity, which might moderate the reciprocal impacts on quality. In particular, considering the analysis of the interactions between the complexity indices, results seem to indicate that large networks in terms of number of authors increase quality only if the institutional complexity is at its minimum. If the variety of institutions is at its maximum, to increase the number of co-authors causes a decrease in the number of citations. On the other hand, in the cases in which the institutional complexity is maximum, i.e., all types of institutions participate in the research in the same proportion, to increase the country complexity allows to increase the number of citations. In other words, we may say that research quality benefits from complex research networks, both in terms of countries and of types of institutions involved, but such networks should be “compact” in terms of number of authors. Vice versa, when the variety of institutions is low, higher results are reached by wider networks in terms of number of authors.

As far as policy design implications are concerned, our first pilot study indicates two main suggestions. First, it is not sufficient to promote the enlargement of research networks per se, given that the different dimensions of complexity might have contrasting impacts on quality. This suggests that to design policies simply aimed at fostering the access to TRN might not be enough to reach better research, at least in the field of gerontology. For projects involving different institutions, there might be multiplier effects on quality if also large TRN are promoted, since the two components of complexity reinforce each other. Second, other scientific fields might give different results, and this implies that—at least in the case of health-related sectors—high caution is needed while designing policy tools aimed at increasing the strength on the national scientific system.

Even if this analysis represents a first exercise limited to a very specific sector and taking into account only publications with at least one author located in China, its relevance is manifold. First of all, to the best of our knowledge, it is the first attempt of taking into account the different dimensions of complexity. This not only allows to identify a possible impact of each component on the quality of research, but it also permits to identify interaction effects among them. Second, the methodology developed in this paper can be extended to other sectors and/or to other countries. As it is reasonable to assume, the impact that complexity has on the quality of the research carried out in a specific country also depends on the “maturity” reached by its scientific system, and cross-country comparisons may shed light on the capacity of each research environment to manage and to exploit the TRN in which it participates. Furthermore, to identify the determinants of research quality may help policy makers in the identification of what to foster and what to facilitate while designing policies aimed at strengthening the competitiveness of the research system of a country.

## Figures and Tables

**Table 1:** Reprint author’s nationality and the role of China.

Number	Total	By Reprint Author		
		Chinese	Foreign	No Reprint Author
Majority	128	91	23	14
Minority	134	23	97	14
Parity	105	40	31	34
Total	367	154	151	62

Source: authors’ elaboration based on ISI WoK data.

**Table 2:** Control variables included in the model.

Name of the Variable	Description
*Nr_Sect*	Number of scientific sectors to which the publication refers
*Repr_NA*	Dummy = 1 if no reprint author is indicated in the publication
*Repr_UNI*	Dummy = 1 if the reprint author’s affiliated institution is a University
*Repr_FOR*	Dummy = 1 if the reprint author is from a foreign institution
*Repr_US*	Dummy = 1 if the reprint author’s country is the US
*ASS*	Dummy = 1 if at least one association takes part in the publication
*COMP*	Dummy = 1 if at least one company takes part in the publication
*HOSP*	Dummy = 1 if at least one hospital takes part in the publication
*PUB*	Dummy = 1 if at least one public institution takes part in the publication
*UNI*	Dummy = 1 if at least one university takes part in the publication
*UNIHOSP*	Dummy = 1 if at least one university-hospital takes part in the publication
*US*	Dummy = 1 if US-based authors take part in the publication
*Impact_Factor*	5-year impact factor (2015) of the journal of the publication ^i^
*CN_repr_maj*	Dummy = 1 if the reprint author is located in China and Chinese institutions represent the majority
*CN_repr_min*	Dummy = 1 if the reprint author is located in China and Chinese institutions represent the minority

^i^ For the journals for which the data on 2015 were not available, the last available previous information has been taken.

**Table 3:** Descriptive statistics of the dependent variable *CIT* (number of citations). Source: authors’ elaboration based on ISI WoK data.

Variable	Mean	Standard Deviation	Variance	Median	Proportion of Zeros
*CIT*	5.605	11.665	136.078	1	0.447

**Table 4:** Regression analysis.

*CIT*	(1)	(2)	(3)
*STD_AUTH*	0.012 **	0.021 **	0.022 ***
	(−2.15)	(2.57)	(−2.64)
*S(c)*	−0.002	−0.015 **	−0.011
	(−0.46)	(−2.12)	(−1.36)
*S(i)*	−0.009	−0.005	−0.005
	(−1.35)	(−0.82)	(−0.82)
*Nr_Sect*	−0.094 **	−0.084 *	−0.088 **
	(−2.15)	(−1.91)	(−2.01)
*Repr_NA*	−5.131 ***	−5.125 ***	−5.153 ***
	(−18.27)	(−18.22)	(−15.59)
*Repr_UNI*	−0.101	−0.114	−0.109
	(−0.95)	(−1.07)	(−1.02)
*Repr_US*	−0.088	−0.056	−0.011
	(−0.41)	(−0.26)	(−0.05)
*Repr_FOR*	0.073	0.120	0.011
	(−0.43)	(0.71)	(0.05)
*US*	0.364 **	0.435 ***	0.385 **
	(2.36)	(2.76)	(2.43)
*ASS*	0.176	0.031	0.078
	(0.68)	(0.11)	(0.27)
*COMP*	0.025	−0.031	−0.035
	(0.08)	(−0.10)	(−0.11)
*HOSP*	0.297	0.258	0.27
	(1.45)	(1.12)	(1.17)
*PUB*	0.272	0.218	0.236
	(1.27)	(0.89)	(0.97)
*UNIHOSP*	0.425 **	0.359	0.364
	(2.01)	(1.49)	(1.51)
*UNI*	0.732 ***	0.744 ***	0.749 ***
	(3.17)	(2.95)	−2.98
S(c)*S(i)		0.000 ***	0.000 ***
		(3.00)	−2.88
STD_AUTH*S(i)		−0.000 *	−0.000 *
		(−1.78)	(−1.80)
STD_AUTH*S(c)		0.004	−0.001
		(0.24)	(−0.07)
*CN_reprint_maj*			−0.013
			(−0.06)
*CN_repr_min*			−0.596 **
			(−2.07)
*Impact_Factor*	0.091 ***	0.089 ***	0.090 ***
	(−4.8)	(4.69)	(4.74)
*Constant*	2.199 ***	2.178 ***	2.188 ***
	(9.08)	(7.44)	(6.29)
*Year dummies*	Yes	Yes	Yes
*N*	1362	1362	1362
*Pseudo R-sq*	0.218	0.219	0.22
*AIC*	5282.493	5278.276	5277.248
*BIC*	5386.828	5398.261	5407.665
*Log-likelihood*	−2621.25	−2616.138	−2613.62

T-statistics in parentheses. Significance: * *p* < 0.1, ** *p* < 0.05, *** *p* < 0.01.

**Table 5:** Average marginal effects.

*CIT*	(1)	(2)	(3)
*STD_AUTH*	0.068 **	0.122 **	0.065 *
*S(c)*	−0.013	−0.089	−0.018
*S(i)*	−0.05	−0.031	−0.04
*Nr_Sect*	−0.546 **	−0.487 ***	−0.51 **
*Repr_NA*	−29.721 ***	−29.739 ***	−29.816 ***
*Repr_UNI*	−0.587	−0.662	−0.632
*Repr_US*	−0.513	−0.323	−0.066
*Repr_FOR*	0.425	0.697	0.063
*US*	2.107 **	2.523 ***	2.229 **
*ASS*	1.017	0.181	0.449
*COMP*	0.145	−0.177	−0.201
*HOSP*	1.722	1.499	1.561
*PUB*	1.574	1.267	1.366
*UNIHOSP*	2.459 **	2.085	2.108
*UNI*	4.239 ***	4.32 ***	4.337 ***
*CN_reprint_maj*			−0.074
*CN_repr_min*			−3.45 **
*Impact_Factor*	0.528 ***	0.516 ***	0.52 ***

Significance: * *p* < 0.1, ** *p* < 0.05, *** *p* < 0.01.

## Appendix A

**Table A1:** Correlation between explanatory variables.

	STD_AUTH	S(c)	S(i)	Nr_Sect	Repr_NA	Repr_UNI	Repr_US	Repr_FOR	CN_Only	US	ASS_Sum	COMP_Sum	UNIHOSP_Sum	UNI_Sum	HOSP_Sum	PUBB_Sum
STD_AUTH	1.000															
S(c)	0.336	1.000														
S(i)	0.380	0.304	1.000													
Nr_Sect	0.078	0.054	0.057	1.000												
Repr_NA	−0.339	−0.229	−0.324	−0.101	1.000											
Repr_UNI	0.152	0.327	0.061	0.123	−0.512	1.000										
Repr_US	0.152	0.378	0.128	0.032	−0.172	0.230	1.000									
Repr_FOR	0.212	0.596	0.228	0.040	−0.262	0.356	0.659	1.000								
CN_Only	−0.209	−0.903	−0.277	−0.034	0.233	−0.326	−0.385	−0.573	1.000							
US	0.169	0.679	0.163	0.019	−0.161	0.226	0.528	0.382	−0.729	1.000						
ASS_Sum	0.165	0.224	0.189	−0.008	−0.055	0.022	0.109	0.142	−0.145	0.148	1.000					
COMP_Sum	0.079	0.117	0.166	−0.011	−0.060	0.059	0.111	0.153	−0.106	0.054	0.019	1.000				
UNIHOSP_Sum	0.302	0.068	0.326	0.012	−0.194	−0.205	0.019	0.068	−0.002	−0.007	0.040	−0.055	1.000			
UNI_Sum	0.477	0.647	0.191	0.026	−0.268	0.487	0.315	0.376	−0.513	0.461	0.180	0.072	−0.082	1.000		
HOSP_Sum	0.244	0.040	0.291	0.071	−0.060	−0.149	−0.015	−0.002	0.044	−0.047	0.006	0.010	−0.061	−0.050	1.000	
PUBB_Sum	0.207	0.229	0.345	−0.009	−0.108	0.113	0.205	0.210	−0.198	0.172	0.050	0.149	0.028	0.195	−0.009	1.000
